# Sorting Transcriptomics Immune Information from Tumor Molecular Features Allows Prediction of Response to Anti-PD1 Therapy in Patients with Advanced Melanoma

**DOI:** 10.3390/ijms24010801

**Published:** 2023-01-02

**Authors:** Lucía Trilla-Fuertes, Angelo Gámez-Pozo, Guillermo Prado-Vázquez, Rocío López-Vacas, Andrea Zapater-Moros, Elena López-Camacho, María I. Lumbreras-Herrera, Virtudes Soriano, Fernando Garicano, Mª José Lecumberri, María Rodríguez de la Borbolla, Margarita Majem, Elisabeth Pérez-Ruiz, María González-Cao, Juana Oramas, Alejandra Magdaleno, Joaquín Fra, Alfonso Martín-Carnicero, Mónica Corral, Teresa Puértolas, Ricardo Ramos, Juan Ángel Fresno Vara, Enrique Espinosa

**Affiliations:** 1 Molecular Oncology Lab, Hospital Universitario La Paz-IdiPAZ, 28046 Madrid, Spain; 2 Biomedica Molecular Medicine SL, 28049 Madrid, Spain; 3 Instituto Valenciano de Oncología, 46009 Valencia, Spain; 4 Spanish Melanoma Group (GEM), 08024 Barcelona, Spain; 5 Hospital de Galdakao, 48960 Bizkaia, Spain; 6 Complejo Hospitalario de Navarra, 31008 Pamplona, Spain; 7 Hospital de Valme, 41014 Sevilla, Spain; 8 Hospital de la Santa Creu i Sant Pau, 08025 Barcelona, Spain; 9 Hospital Costa del Sol, 29603 Marbella, Spain; 10 Hospital Quirón Dexeus, 08028 Barcelona, Spain; 11 Hospital Universitario de Canarias-San Cristóbal de la Laguna, 38320 La Laguna, Spain; 12 Hospital Universitario de Elche y Vega Baja, 03203 Elche, Spain; 13 Hospital Universitario Río Hortega, 47012 Valladolid, Spain; 14 Hospital San Pedro, 26006 Logroño, Spain; 15 Hospital Clínico Lozano Blesa, 50009 Zaragoza, Spain; 16 Hospital Universitario Miguel Servet, 50009 Zaragoza, Spain; 17 Genomics Unit, Parque Científico de Madrid, 28049 Madrid, Spain; 18 CIBERONC, ISCIII, 28029 Madrid, Spain; 19 Medical Oncology Service, Hospital Universitario La Paz, 28046 Madrid, Spain

**Keywords:** melanoma, immunotherapy, prediction of response, computational analysis, personalized medicine

## Abstract

Immunotherapy based on anti-PD1 antibodies has improved the outcome of advanced melanoma. However, prediction of response to immunotherapy remains an unmet need in the field. Tumor PD-L1 expression, mutational burden, gene profiles and microbiome profiles have been proposed as potential markers but are not used in clinical practice. Probabilistic graphical models and classificatory algorithms were used to classify melanoma tumor samples from a TCGA cohort. A cohort of patients with advanced melanoma treated with PD-1 inhibitors was also analyzed. We established that gene expression data can be grouped in two different layers of information: immune and molecular. In the TCGA, the molecular classification provided information on processes such as epidermis development and keratinization, melanogenesis, and extracellular space and membrane. The immune layer classification was able to distinguish between responders and non-responders to immunotherapy in an independent series of patients with advanced melanoma treated with PD-1 inhibitors. We established that the immune information is independent than molecular features of the tumors in melanoma TCGA cohort, and an immune classification of these tumors was established. This immune classification was capable to determine what patients are going to respond to immunotherapy in a new cohort of patients with advanced melanoma treated with PD-1 inhibitors Therefore, this immune signature could be useful to the clinicians to identify those patients who will respond to immunotherapy.

## 1. Introduction

Melanoma is the most lethal cutaneous cancer, with over 7000 estimated deaths in the United States in 2021 [1]. Early detection allows a curative resection, but once the tumor disseminated the prognosis is poor [2].

The relationship between melanoma and the immune system is well established and justifies the use of immunotherapy to treat advanced disease. Interferon alpha and interleukin-2 produced occasional responses, although at the cost of high toxicity [3]. More recently, immune therapy with anti-CTLA4 and anti-PD1 antibodies has become standard of care [4,5,6]. Some patients obtain long-term benefit with anti-PD1 based therapy, whereas others with a similar clinical background have an early progression. Prediction of response to these drugs remains an unmet need in the field. Markers such as PD-L1 expression, tumor mutational burden and microbiota analysis have been proposed to predict response [7,8]. However, they are not used in clinical practice due to lack of accuracy.

New computational methods for the analysis of genomic information can unravel molecular features related to response to immunotherapy. For instance, probabilistic graphical models (PGM) can be used to build functional networks [9,10]. This kind of networks provide information about relevant processes taking place in the tumor cell.

In this study, RNA-seq data from melanoma samples were analyzed through PGM and classificatory algorithms to define relevant molecular processes in the tumors and to search for molecular mechanisms related to resistance and sensitivity to immunotherapy.

## 2. Results

### 2.1. TCGA Cohort

TCGA RNA-seq data included 472 samples. One duplicated and one normal tissue samples were removed. Twenty-five patients had received neoadjuvant treatment so their corresponding samples were also excluded. The final number of samples used for the subsequent analyses were 446. Additionally, there were two patients with a paired primary tumor-metastasis samples, so the final number of patients was 444. Clinical characteristics were summarized in Supplementary Table S1. 183 samples had a mutation in BRAF, 88 a mutation in NRAS, 24 a mutation in NF1, 54 were triple negative (TN) and 95 had not information about their biomarker status. The median follow-up was 395 days, and 143 deaths had occurred.

### 2.2. Functional Characterization of the TCGA Cohort

**Figure 1:**
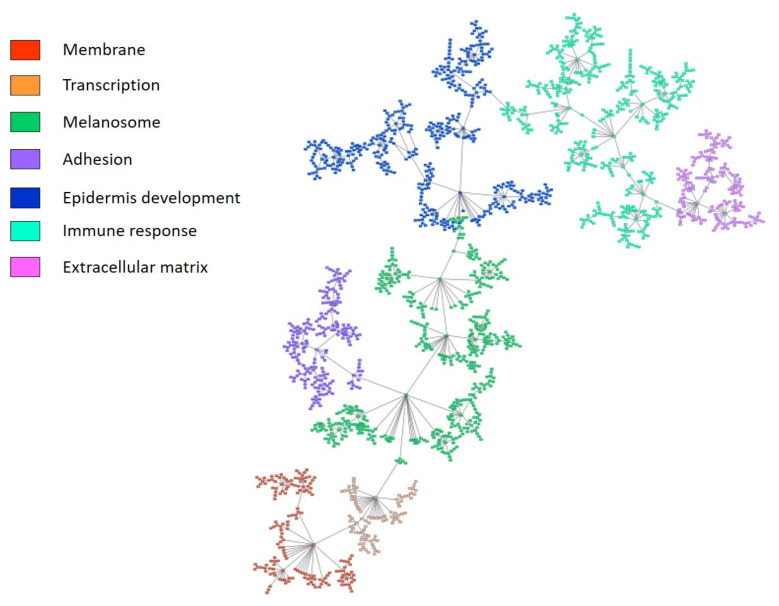
Probabilistic graphical model built using the 2000 most variable genes from TCGA melanoma cohort. The network was divided into seven functional nodes.

PGM was built using the 2000 most variable genes. The resulting graph was processed to seek for functional structure. Overall, we divided the obtained network into seven functional nodes with an overrepresented biological function (Figure 1, Supplementary Table S2).

### 2.3. Functional Differences According to Mutational Subtypes in the TCGA Cohort

We explored if the mutational status of the tumors correlated with differences in the activity of functional nodes (Supplementary Figure S1). There were significant differences between BRAF positive and TN, and NRAS positive and TN in the activity of the nodes “membrane”, “melanosome” and “adhesion”.

### 2.4. Biological Layer Analysis

We applied a recursive sparse k-means/Consensus cluster algorithm (CCA) workflow, using the 2000 most variable genes as showed in previous works [11,12]. We defined seven different layers with diverse molecular functions, such as melanogenesis, immune, epidermis development, extracellular space, and inflammatory response. All layers were divided into two groups except layers 1 and 3, which were divided into three and four groups respectively. Results are summarized in Table 1.

#### 2.4.1. Layer 1: Melanogenesis

The layer “melanogenesis” included 57 genes mainly related with the melanogenesis process, for instance, MLANA, TYR, TYRP1, OCA2, DCT, GPR143, and SLC45A2. The layer was divided into three clusters including 219 (49%), 164 (36.7%) and 64 (14.3%) patients respectively. Functional node activity analyses showed a decreased activity of the melanosome node through clusters, being higher in cluster 1.1 and lower in cluster 1.3. Cluster 1 tumors also showed lower activity of the nodes “membrane”, “translation”, “adhesion”, “extracellular matrix”, “immune” and “epidermis development” when compared with clusters 2 and 3. Clusters 2 tumors showed lower activity in the nodes “translation” and “extracellular space” when compared with cluster 3 tumors (Supplementary Figure S2).

#### 2.4.2. Layer 2: Immune Response

**Figure 2:**
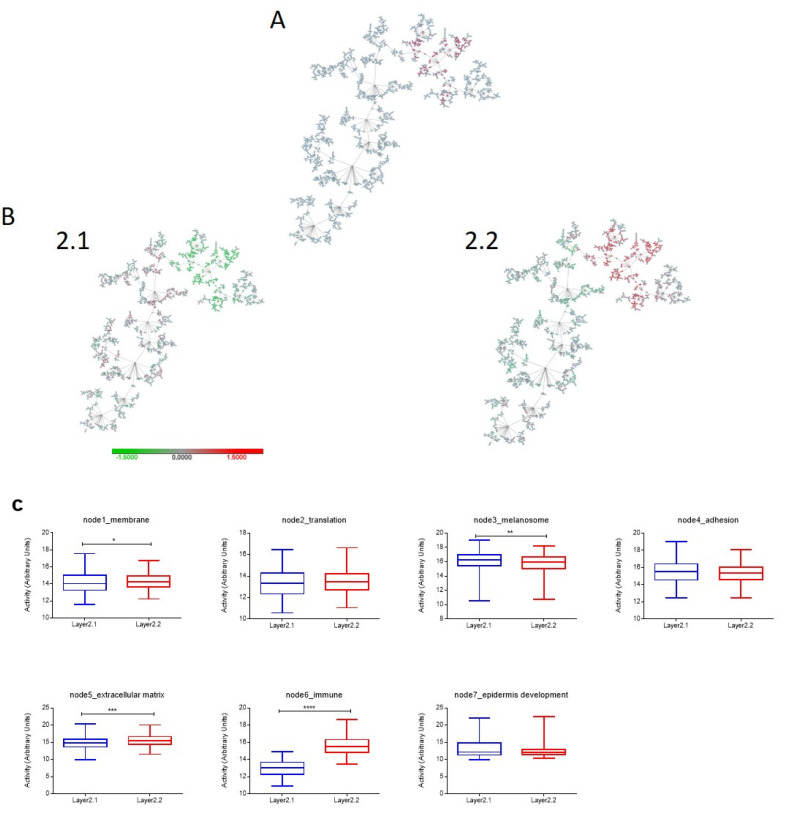
Second layer reflecting immune information in TCGA melanoma cohort. (**A**) Location of the 146 genes defining Layer 2 in the network. (**B**) Heatmap network of Layer 2 groups. (**C**) Comparison of functional node activities between defined groups. ****, *p* ≤ 0.0001; ***, *p* ≤ 0.001; **, *p* ≤ 0.01; *, *p* ≤ 0.05.

The second layer included 146 genes mainly related to the immune response. Cluster 2.1 had 221 patients (49%) and was characterized by a low activity of immune node, so we called it “Immune-low group”. Cluster 2.2 included 226 patients (51%) and was characterized by high functional activity of the node, so we renamed it as “Immune-high group” (Figure 2). Immune genes in this biological layer include mainly cytokines and chemokines and other relevant immune genes such as FASL, ICOS, IFNG, CD8A, and CD8B.

#### 2.4.3. Layer 3: Epidermis Development & Keratinization

This layer included 63 genes, mainly related to epidermis development and keratinization such as keratins (KRT17, KRT15, KRT14, KRT5, KRT6A, and KRT6B) and other relevant genes such as COL17A1, GRHL3, or DSP. Four groups were established by CCA (Table 1, Supplementary Figure S3). Cluster 3.2 showed the lowest activity of the node, followed by 3.3, then 3.1 and, finally, 3.4 (highest functional node activity).

#### 2.4.4. Layer 4: Extracellular Space & Membrane

The fourth layer was based on 86 genes mainly related with extracellular matrix and adhesion, such as PTN, SERPINA5, LPAR1, PHKA1, or CLDN11. Cluster 4.1 included 236 patients (53%), with lower functional activity of the nodes “extracellular matrix” and “adhesion”, whereas Layer 4.2 included 211 patients (47%), with higher functional activity of these nodes (Supplementary Figure S4).

#### 2.4.5. Layers 5, 6 and 7

Layer 5 grouped patients again according to genes related to extracellular matrix and adhesion, and Layer 6 was associated with inflammation and immune response. Finally, Layer 7 showed no overrepresented function. Therefore, we decided to stop the layer analysis at this point, because functional information was already redundant (Table 1).

In order to visualize the different classifications defined, a hierarchical cluster (HCL) was constructed (Supplementary Figure S5).

### 2.5. Immune Classification

**Figure 3:**
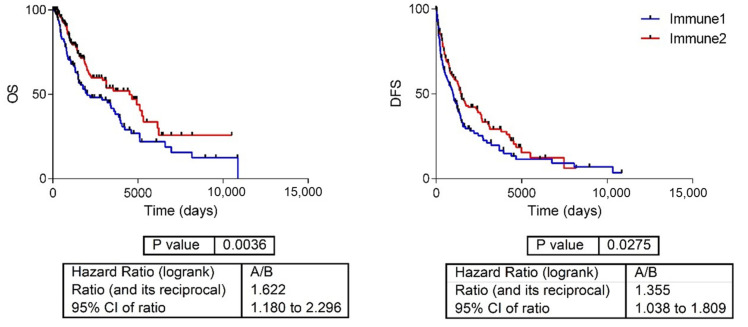
Survival curves according immune classification in the TCGA cohort. OS = overall survival. DFS = disease-free survival.

Layers 2 and 6, both related to immune information, supplied overlapping classifications. So, Layer 2 was considered as the “immune classification” in the TCGA series. This layer provided prognostic information regarding overall survival (OS) and disease-free survival (DFS) (Figure 3).

### 2.6. Molecular Classification

**Figure 4:**
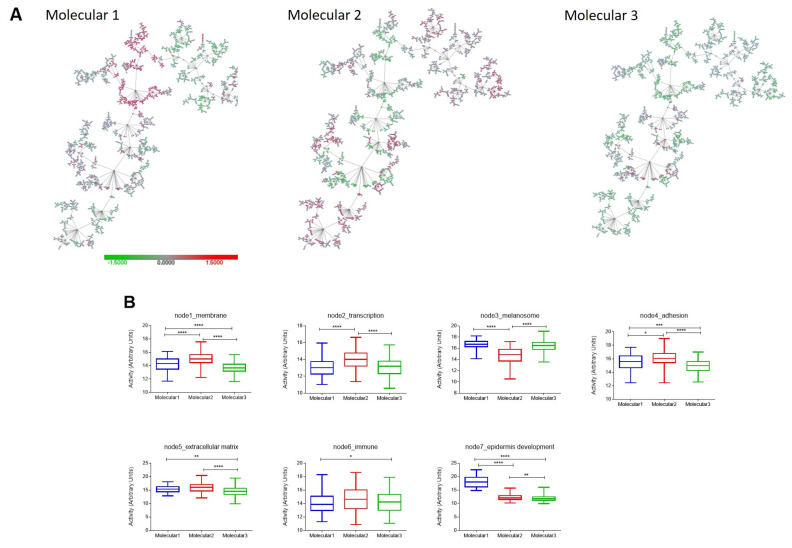
Molecular classification grouping information of molecular layers defined in the TCGA melanoma cohort. (**A**) Groups obtained using molecular information. (**B**) Functional node activities in molecular groups. ****, *p* ≤ 0.0001; ***, *p* ≤ 0.001; **, *p* ≤ 0.01; *, *p* ≤ 0.05.

Layers 1, 3 and 4 contained molecular information about biological functions such as melanogenesis, cellular adhesion and keratinization. None of these molecular layers had a significant association with prognosis (Supplementary Figure S6). These layers showed partial overlapping in the HCL and, therefore, they were grouped into a unique informative layer renamed “molecular classification”, and three new groups were established using 206 genes obtained from these molecular layers (Figure 4A).

Molecular 1, which included 70 patients (16%), was characterized by higher activity of the functional node “epidermis development” and lower activity of the node “membrane”. Molecular 2, with 154 patients (34%), had the highest activity of the nodes “membrane”, “transcription” and “adhesion”; and the lower activity of the node “melanosome”. Finally, Molecular 3 included 223 patients (50%) and was characterized by lower activity of the node “extracellular matrix” and higher activity of the node “melanosome” (Figure 4B). The activity of the immune node was residual in the three groups, suggesting that the immune and molecular classifications are independent.

A Significance Analysis of Microarrays (SAM) among the three molecular groups identified 170 out of 206 genes defined by layer analysis as differentially expressed (Supplementary Figure S7). Genes exclusively overexpressed in Molecular 1 included keratins (KRT80, KRT15, KRT14, KRT6A, KRT5, KRT6B, KRT17) and other genes related to epidermis development such as DSP, COL17A1, CDSN, GRHL3, CST6, EVPL, GJB5, ZNF750, or SPRR2D. Some of the genes overexpressed in Molecular 1 and Molecular 3 included MLANA, TYR, TYRP1, OCA2, DCT, GPR143, and SLC45A2, all of them involved in melanogenesis. Finally, Molecular 2 overexpressed genes related to extracellular region were PCSK1, SEMA3D, CFI, LOXL4, SEMA3E, PTN, FGF2, SORL1, F5, SERPINA5, SFRP1, FLRT3, GPC4, ACPP, and ANGPTL1. This group also overexpressed genes related to plasma membrane such as LPAR1, TMEM100, STRA6, KCNIP1, TESC, PHKA1, LIFR, GFRA1, CLDN11, ITGA10, and CNTN4.

As it was seen in the analysis of molecular layers, none of these molecular groups correlated with DFS or OS (Supplementary Figure S8).

Interestingly, the distribution between primary tumors and metastases varied between molecular groups, primary tumors being mainly included in Molecular 1 (Supplementary Figure S9).

### 2.7. Cohort of Patients Treated with Anti-PD1 Therapy in the Spanish Melanoma Group (GEM)

Fifty-two patients with advanced melanoma receiving anti-PD1 antibodies were recruited for this study by the Spanish Melanoma Group (GEM). The series included 26 primary tumor samples, 10 lymph nodes, and 16 metastases. Median follow-up was 12.6 months. Eleven patients achieved a complete response, 13 a partial response, 10 stable disease, and 13 had a progression. Clinical data of this cohort is summarized in Supplementary Table S3.

### 2.8. Sample Processing and RNA Capture Experiments

Fifty-two paraffin samples were retrieved, although four of them did not yield enough material to perform RNA extraction. After RNA extraction, eight samples were excluded due to low RNA quantity yield. Therefore, 40 samples were analyzed by RNA-seq. 2268 genes presented more than 400 lectures across the 40 patients and 2151 genes had less than 50% of zeroes.

### 2.9. Biological Layer Classification in GEM Cohort

With the aim of assessing the robustness of the immune and molecular classifications previously described in the TCGA series, patients from the GEM cohort were classified by CCA using the genes defined as most important in each layer classification. The number of groups was consistent between GEM and TCGA cohorts, with the exception of Layer 3 (Epidermis development and keratinization). In TCGA cohort 4 groups were defined in this layer whereas in GEM cohort, only two groups were identified (Supplementary Table S4).

### 2.10. Relation to Survival of Molecular and Immune Classifications in GEM Cohort

With the aim of studying the influence of PD-1 inhibitors in the molecular and immune characterization of advanced melanoma, survival comparisons were performed between the defined groups. In this case, the two groups defined in Layer3 (Epidermis development and keratinization) had significantly different OS (*p* = 0.03, HR = 3.55) (Supplementary Figure S10).

Again, the molecular classification differentially classified primary tumors and metastases (Supplementary Figure S11).

**Figure 5:**
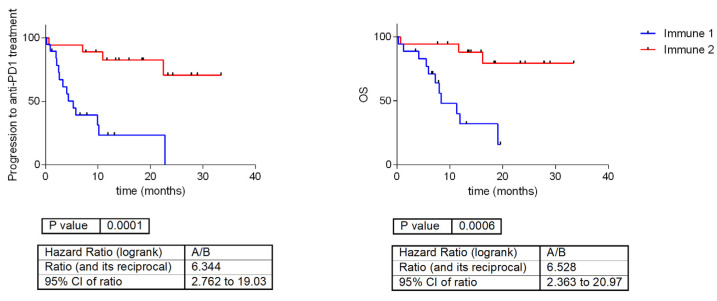
Survival curves according immune information previously defined in TCGA cohort in GEM cohort treated with anti-PD1 inhibitors. Immune 1 is the “immune-low group”, showing lower expression of immune-related genes, and Immune 2 is the “immune-high group”, presenting higher expression of immune-related genes. OS = overall survival.

The immune information defined in the TCGA cohort had significant prognostic value in the GEM cohort treated with PD-1 inhibitors. The “immune-low” group had a poor outcome as compared with the “immune-high” group (PFS: *p* = 0.0001, HR = 6.34; OS: *p* = 0.0006, HR = 6.52) (Figure 5).

Of the 146 genes included in this immune layer, 33 had an ontology of “immune response”, mainly cytokines and chemokines. CCL19, CCL21, CCL5, CCR2, CCR7, CD27, CD7, CD79A, CD8A, CD8B, CD96, CR2, CXCL10, CXCL11, CXCL13, CXCL9, FASL, GZMA, ICOS, IFNG, IL2RG, LAX1, LTA, LTB, NCR3, TLR10, and TNFRSF9, all of them showing higher expression in the “Immune-high” group (Supplementary Figure S12).

A summary about the analysis workflow and the main results obtained in this study was showed in Supplementary Figure S13.

## 3. Discussion

In the present study, an approach based on the existence of different informative layers in gene expression and PGM was used to establish immune and molecular subgroups in melanoma samples. Two independent datasets -one coming from TCGA and one from the GEM- were used to test the performance of the method. Genes were classified into functional nodes with specific functions, establishing subgroups based on molecular and immune features. Subgroups related to immunity predicted response to anti-PD1 therapy in the GEM series.

PGM coupled with gene ontology allows studying differences in biological processes, not relying on the expression of one specific gene, but rather on all grouped genes involved in the same process [9,10]. We have previously demonstrated the validity of this approach in breast cancer and bladder cancer [11,12].

Two new classifications of melanoma samples were established by combining PGM, sparse k-means and CCA. The novelty of this approach is that information provided by gene expression is treated as if different informative layers existed, segregating immune and molecular information. TCGA had previously defined melanoma molecular subgroups [13], some of which match with our groups. The TCGA keratin group coincides with our Molecular 1 group, whereas the MITF-low group corresponds to our Molecular 2 group. We identified a new “melanosome high” group (Molecular 3). In a previous reanalysis of TCGA data, Netanely et al. pointed out the relevance of melanosome-related genes [14]. The distinction between immune and molecular information, however, has not been previously described.

Molecular 1 is characterized by high expression of genes related to epidermis development and keratinization. COL17A1 was associated with pigmentation and melanocyte supply to the epidermis [15], and its accumulation in melanocytic tumors has been associated with malignant transformation, having been proposed as a potential target for antibody-induced melanoma apoptosis [16]. GRLH3 is a member of the grainyhead family of transcription factors and a low expression of this protein has been described in non-melanoma skin tumors [17]. Another relevant gene in Molecular 1 group is ZNF750: its overexpression decreases proliferation of melanoma cells, whereas its depletion causes opposite effects [18].Molecular 1 comprised most of primary tumors included in the TCGA and GEM cohorts. The group showed higher activity of the nodes “epidermis development” and “keratin”. This observation agrees with that by Netanely et al. [14].

Molecular 1 and 3 overexpressed genes related to melanogenesis. MLANA and tyrosinase have been proposed as biomarkers for melanoma detection [19,20]. Tyrosinase related protein 1 (TYRP1) has been associated with prognosis and survival [21,22]. Other relevant genes in these groups were the OCA2-melanosomal transmembrane protein (OCA2) [23] and dopachrome tautomerase (DCT) [24].

Molecular 2 overexpressed genes related to adhesion and extracellular matrix. Pleiotrophin (PTN) is a heparin-binding growth factor of the family of midkine, a tumor promoting factor [25,26]. FGF2 plays an important role in melanoma progression and antibodies blocking FGF2 have been proposed as a therapy in metastatic melanoma [27]. LPAR1 is critical in resistance acquisition to BRAF inhibitors [28]. LIFR is associated with unfavorable prognosis [29].

Several molecular groups in melanoma have been defined, even immune clusters have been established [30]. Despite this, they have no clinical utility or relation to prognosis However, our immune classification provides information about response to immunotherapy treatment and prognosis. In the last years, literature has been centered in the study of single genes or in a combination of well-known immune genes to predict response to immunotherapy, but these studies have had a moderate impact in clinical practice [31,32,33]. Our approach offers an integrative vision of many genes related to the immune response and has the extra advantage that is a non-directed analysis, without a priori information, which makes its capable to suggest new hypothesis and relevant biomarkers. In this case, an immune signature was defined based only in the expression data measured in our series, without the need of any a priori knowledge, meaning that these genes were blindly included into the immune classification. This immune signature had a good predictive value and in the future, it could be possible to study the role of the genes that composed this immune signature in response to immunotherapy and also their role in immune response.

The increasing use of immunotherapy in melanoma has highlighted the relevance of the immune status in the outcome of neoplastic diseases. Our immune classification identified an “immune-high” group of patients who responded to anti-PD1 therapy, and an “immune-low” group showing poor response. Our immune signature was mainly formed by cytokines and chemokines, such as CXCL9, CXCR5, CXCL13, CCL5, CCR2 and CCR7 among others, and other immune related genes such as ICOS, CD96, TNFRSF9, IFNG, CD8A, and CD8B. All these genes had higher expression in the “immune-high” group. Some of these genes (CXCL9, CXCL13, CCL5, CD96, TNFRSF9, IFNG, CD8A, and CD8B) have been previously reported by a previous work by Gide et al. where the authors established immune biomarkers related to response to PD1 monotherapy and to the combination of PD1 and CTLA-4 immunotherapy [34]. ICOS (inducible T cell costimulator) belongs to the CTLA-4 protein family. Engagement of ICOS pathway significantly enhances the efficacy of CTLA-4 blockade [35]. Interestingly, Gide et al. identified this immune biomarker both in PD-1 positive and PD-1 negative tumors, proposing it as an alternative immune target [34]. However, in our cohort we found an overexpression of ICOS in the “Immune high” responder group. CCR2 is another chemokine included in our immune signature. It has been described that CCR2 antagonists in combination with anti-PD1 therapy leads to sensitization to anti-PD1 monotherapy in bladder carcinoma murine models [33]. This drug combination should be further explored in melanoma as well.

Cytokines and chemokines have a pro-inflammatory role. Cytokines send intracellular signals by binding to specific surface receptors and they could be involved in cell activation, apoptosis, division or movement [36]. Chemokines are members of the cytokine family and have a key role in leukocyte migration [37]. IFNG (interferon gamma), also included in our immune signature, is only produced by immune cells and activates macrophage and neutrophil intracellular killing, stimulates natural killer cell function and enhances antigen presentation through increasing MHC class II expression on antigen presenting cells [36]. These data suggest that an activation of innate immune response is taking place in the “immune-high” group that favors response to immunotherapy.

This immune signature, defined in TCGA cohort and based on intrinsic immune features, identified patients who responded to immunotherapy in the GEM cohort treated with PD-1 inhibitors. Prediction of response to immunotherapy remains an unmet need in the field of advanced melanoma. The expression of PD-L1 in the tumor has a poor correlation with response, so better markers are urgently needed. Mutational burden, gene profiles and microbiome profiles have been proposed but are not used in clinical practice [7,8]. Molecular signatures are already trying to apply to improve patient management. The so-called interferon-gamma signature has recently been proposed to select immunotherapy in the neoadjuvant setting: tumors with a high baseline signature seem to have a good response to anti-PD1 therapy alone, whereas tumors with a low signature would require combination therapy [38].

The identification of patients with advanced melanoma who will not respond to immunotherapy would have a major impact in their management. Likewise, a predictive tool could also be used in the adjuvant setting, where patients do not derive benefit if they have tumors with primary resistance to immunotherapy. Our immune signature allows determining those patients that will respond to anti-PD1 therapy in an adjuvant context, being able to help to decide which melanoma patients should receive adjuvant anti-PD1 immunotherapy. An accurate marker would allow to spare the side effects and the cost of anti-PD1 antibodies and also opens the possibility to identify patients that will not respond to anti-PD1 therapy and redirected them to a clinical trial in early stages.

Our immune classification had a prognostic value in melanoma TCGA cohort, with the immune-high group showing a trend for a better survival (OS *p* = 0.003, HR = 1.6). Interestingly, in the GEM cohort treated with immunotherapy, the difference in OS between the “immune low” and “immune high” was more striking, (*p* = 0.0006, HR = 6.52), which suggest that the immune signature provides rather predictive information.

Our study has some limitations. First, the TCGA cohort is heterogenous and does not include patients treated with anti-PD1 therapy. This was addressed in our second cohort. However, this clinical cohort was small and also comprised samples from primary tumors as well as metastases. Additionally, no patient received combination immunotherapy with anti-PD1 and anti-CTLA4 antibodies, which is one of the current standard therapies. For this reason, the results should be validated in a larger contemporaneous series.

## 4. Materials and Methods

Two data sets were used, the first coming from The Cancer Genome Atlas (TCGA). These samples come from patients treated before the era of anti-PD1 therapy. The second set came from patients receiving anti-PD1 therapy in a recent clinical series.

### 4.1. Preprocessing of TCGA Melanoma Data

Gene expression data from 472 melanoma tumor samples included in TCGA were obtained by RNA-seq [13]. Genes with an official symbol were selected among a total of 20,500 genes. Data was log2 transformed, then genes with at least 75% of valid values were selected, and missing values were imputed according to a normal distribution in Perseus software using default settings [39]. Finally, genes were ranked according to their standard deviation (SD), and the 2000 genes showing the highest DE were selected for subsequent analyses.

### 4.2. GEM Cohort of Advanced Melanoma Patients Treated with Anti-PD1 Inhibitors

Fifty-two formalin-fixed, paraffin-embedded (FFPE) samples from 52 patients with advanced melanoma and treated with anti-PD1 inhibitors were recruited by the Spanish Group of Melanoma (GEM) for this study.

### 4.3. RNA Isolation

Five to ten 10–15 µm FFPE sections were obtained for each sample. Total RNA was isolated using miRNeasy FFPE Kit (Qiagen) following manufacturer’s instructions. Purified nucleic acid quality control for quantity and purity was assessed using an ND-1000 NanoDrop spectrophotometer (Thermo Fisher Scientific, Waltham, MA, USA).

### 4.4. RNA Capture and Sequencing

100 ng of RNA from each sample were used for library preparation with the KAPA RNA Hyperprep kit (Roche Nimblegen Inc., Pleasanton, CA, USA) following manufacturer’s instructions. Library fragments distribution was confirmed by electrophoresis and concentration was determined using the KAPA library Quantification kit (Roche Nimblegen Inc.). A seven MB SeqCap EZ probe pool (Roche), including genes previously defined, was designed using the NimbleDesign online tool. An equal mass of eight cDNA libraries was pooled and hybridized with the SeqCap EZ probe pool following manufacturers’ specifications. Samples were sequenced in two groups using pair end 2 × 100 NextSeq 50/550 high Output Cartridge v2, 75 cycles. Mapping with TopHat and FPKM calculation using CuffLinks was performed using the G-Pro Suite [40].

### 4.5. Preprocessing of RNA Capture Data

First, ensembl gene notation was translated to official gene symbol using ensembl Biomart tool [41]. Seven gene symbols were duplicated, so the normalized counts of these genes were added to each other. Those genes with at least 400 counts in the 40 analyzed samples were selected. Data was log2 transformed and those genes with more than 50% of zeroes were removed. Finally, missing values imputation according to a normal distribution was performed using Perseus.

### 4.6. Network Construction and Functional Node Activities

Functional networks were built using probabilistic graphical models (PGM) and expression data without other a priori information, as previously described [9,10], using *grapHD* [42] and R environment. PGMs with high-dimensionality that minimize Bayesian Information Criterion (BIC) were used. These PGMs are based on two sequential steps: first, the spanning tree with the maximum likelihood is established, and, second, a forward search for adding edges that minimizes BIC and preserve graph decomposability is done [43]. With the aim of establish a functional structure, the networks obtained from PGMs were split into several branches and gene ontology analyses were made in order to determine the main function of each branch. Gene ontology analyses were performed with DAVID webtool using “Homo sapiens” as background and GOTERM-FAT, KEGG and Biocarta categories.

To perform this analysis, 2000 more variable genes according to their standard deviation (SD) from TCGA cohort were used. Then, functional node activities were calculated by the mean expression of genes/proteins of each node related with its main function.

### 4.7. Biological Informative Layer Analyses

In order to characterize groups in TCGA melanoma tumors, sparse k-means and Consensus Cluster algorithm (CCA) were used as previously shown [11,12]. Briefly, sparse K-means was used to select and weight variables based on their relevance in sample classification [44], followed by a Consensus Clustering algorithm (CCA) analysis, that provides an optimum number of clusters for each classification [45]. First, sparse k-means identified a set of genes that are relevant to classify the tumors, and, then, CCA established the optimum number of groups in which the population should be divided.

To explore the existence of different informative layers, sparse k-means and CCA were performed successive times. After establishing relevant genes for one classification, these genes were removed from the dataset and a sparse k-means and CCA were done again using the remaining genes. The method allows to identify different layers of information and establish different classifications based on various features. Gene ontology analyses were used to establish the type of information provided by each layer.

Hierarchical cluster (HCL) was used to evaluate differences in these classifications. In the case of immune layers, established classifications seemed to be equivalent to each other. On the contrary, layers containing molecular information showed non overlapping clusters, providing complementary information. So, we considered separately the immune information and the other molecular layers. A CCA including all genes for molecular layers was performed, resulting in a definitive classification based on molecular information.

Samples from the GEM melanoma cohort were evaluated for each classification defined in the TCGA cohort, using the genes selected by sparse k-means and a new CCA for each classification.

### 4.8. Statistical Analyses

Statistical analyses were performed in GraphPad Prism v6. Differences in functional node activities between groups were evaluated using Mann-Whitney and Kruskal-Wallis test. Survival analyses were done using a Kaplan Meier test. Cytoscape software was used for network visualization Hierarchical clusters and Significance Analysis of Microarrays (SAM) was done using MeV. SAM identified those genes differentially expressed between predefined groups by a t test corrected by the number of samples [46]. All *p*-values were two sided and *p* < 0.05 was considered statistically significant.

## 5. Conclusions

In conclusion, we established that the immune information was independent from tumor molecular features in melanomas included in the TCGA cohort. An immune and a molecular classification (based on melanogenesis, adhesion and epidermis development) of these tumors was established. Remarkably, the immune classification predicted response to immunotherapy in a cohort of patients with advanced melanoma treated with PD-1 inhibitors. This immune signature could be used by the clinicians to identify patients who will respond to immunotherapy.

## Figures and Tables

**Table 1:** Summary of defined biological layers. CCA: Number of groups determined by consensus cluster algorithm.

				Cluster Distribution%			
Layer	Main Function	CCA	Genes	1	2	3	4
1	Melanogenesis	3	57	49	37	14	--
2	Immune	2	146	49	51	--	--
3	Epidermis development & keratinization	4	63	10.8	57.7	21.9	9.6
4	Extracellular space & membrane	2	86	53	47	--	--
5	Extracellular space & extracellular matrix	2	110	66	34	--	--
6	Inflammatory response	2	125	49	51	--	--
7	Without function	2	11	41	59	--	--

## Data Availability

RNA-seq data is publicly available at https://www.ebi.ac.uk/arrayexpress/, accessed on 7 November 2022, under the identification E-MTAB-11729.

## Supplementary Materials

The following supporting information can be downloaded at: https://www.mdpi.com/article/10.3390/ijms24010801/s1.
