# Supplementary material for: Sorting Transcriptomics Immune Information from Tumor Molecular Features Allows Prediction of Response to Anti-PD1 Therapy in Patients with Advanced Melanoma
Source: Int J Mol Sci. 2023 Jan 2;24(1):801. doi: 10.3390/ijms24010801 (PMC9821399; doi:10.3390/ijms24010801)
Supplement: Supplementary file 1 [file ijms-24-00801-s001.zip › Tables S1, S3 and S4 and Figures S1-S12.pdf]

Table S1: Patient's characteristics of TCGA melanoma cohort.

	Number of patients	Percentage
**Number of patients**	444	100%
**Age at diagnosis (median and range)**	58 (18-90)	
**Age at diagnosis (mean)**	58	
**Gender**		
**Male**	274	62%
**Female**	169	38%
**Ulceration**		
**Yes**	161	36.3%
**No**	139	31.3%
**Unknown**	144	32.4%
**Lymph node status**		
**N0**	223	50.2%
**N positive**	202	45.5%
**Unknown**	19	4.3%
**TNM stage AJCC**		
**0**	6	1.4%
**I**	75	17%
**II**	133	30%
**I/II**	12	2.7%
**III**	160	36%
**IV**	23	5%
**Unknown**	35	7.9%

Table S3: Patients' characteristics of Spanish Melanoma Group (GEM) advanced melanoma cohort treated with PD-1 inhibitors.

	Number of patients	Percentage
**Number of patients**	52	100%
**Age at diagnosis (median and range)**	66 (33-88)	
**Age at diagnosis (mean)**	64	
**Gender**		
**Male**	35	67.3%
**Female**	17	32.6%
**BRAF**		
**Positive**	15	28.8%
**Negative**	29	55.8%
**Unknown**	8	15.4%
**Anti-PD1 treatment**		
**Pembrolizumab**	27	52%
**Nivolumab**	25	48%

Table S4: Layer classifications in GEM cohort.

Layer	Function	Groups
1	Melanogenesis	3
2	Immune response	2
3	Epidermis development & keratinization	2
4	Extracellular space & membrane	2

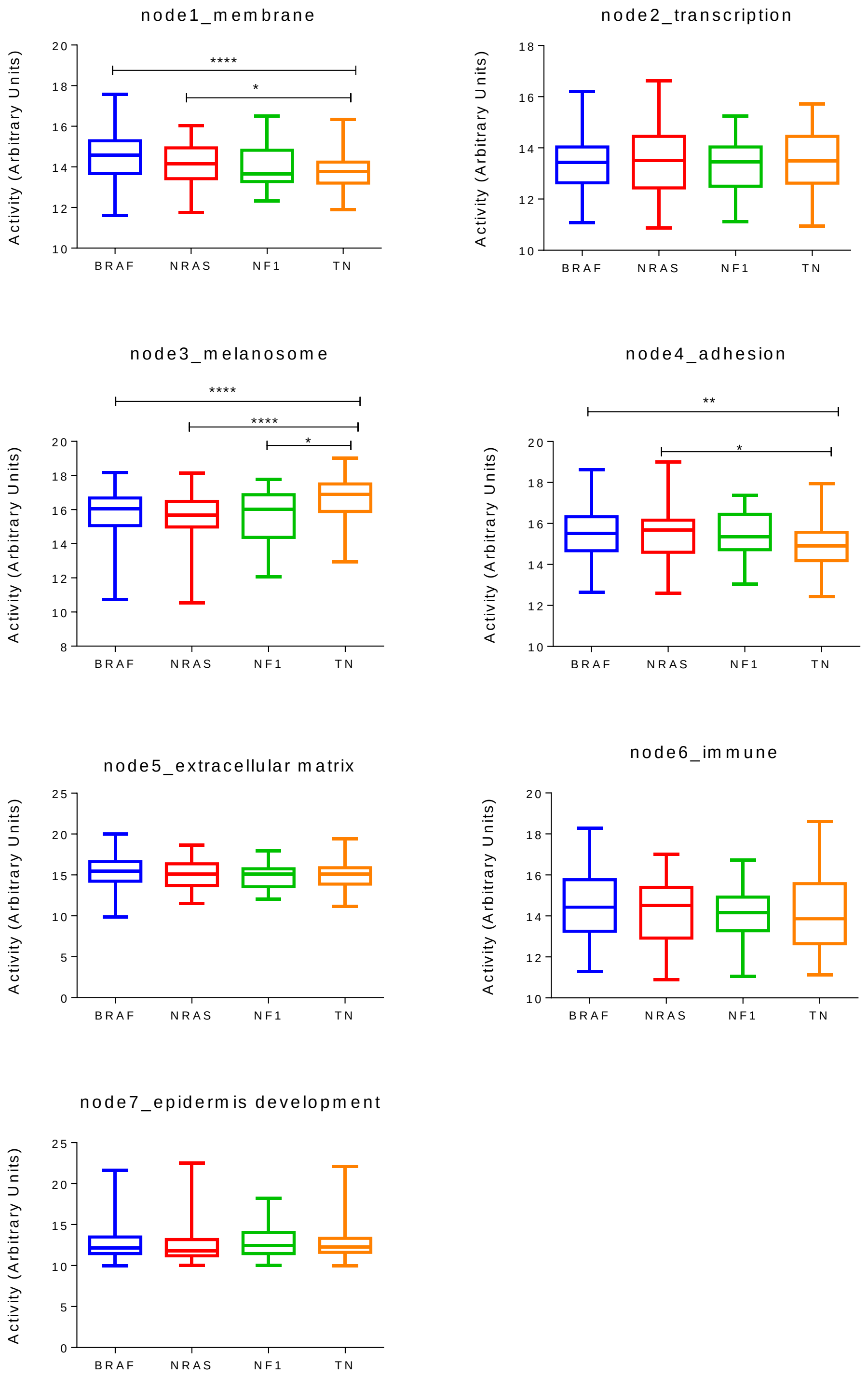

Figure S1: Node activity measurements according to mutational status in TCGA melanoma cohort. ****, ≤ 0.0001; ***, ≤ 0.001; **, ≤ 0.01; * ≤ 0.05. BRAF = BRAF-mutant positive, NRAS= NRAS-mutant, NF1= NF1-mutant, TN= triple negative.

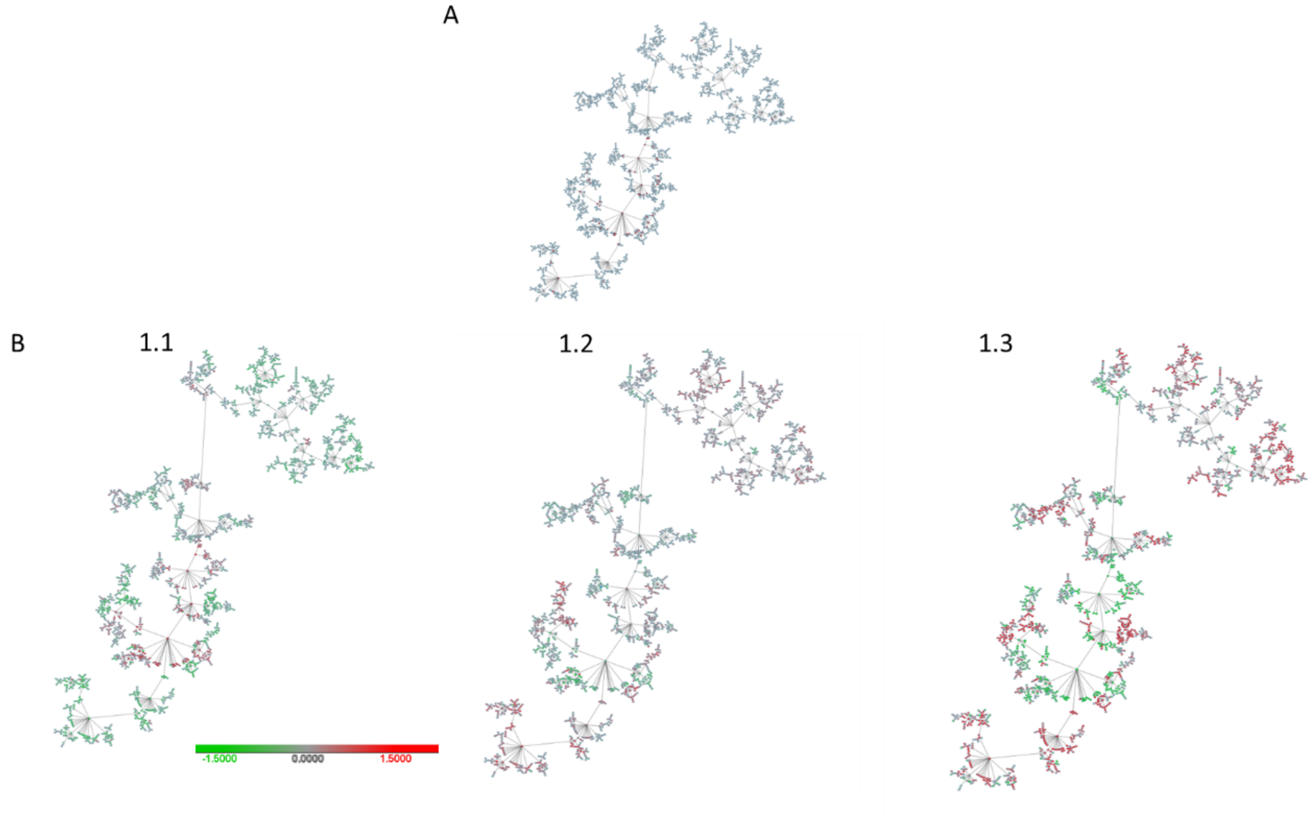

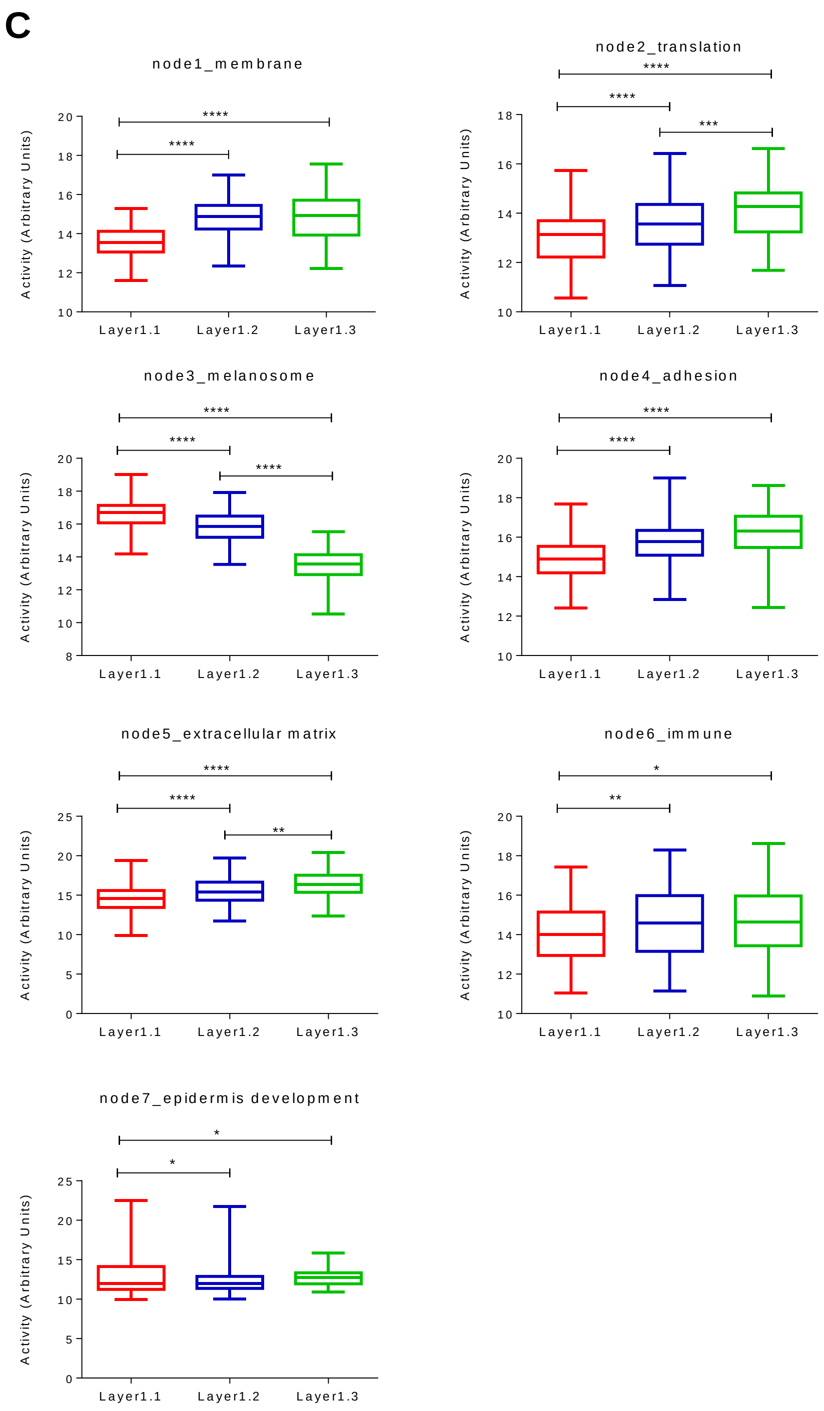

Figure S2: First layer reflects melanogenesis information in TCGA melanoma cohort. A) Network location of the 57 genes defining Layer 1. B) Heatmap networks of the three groups of Layer 1. C) Comparison of functional node activities between defined groups. ****, ≤ 0.0001; ***, ≤ 0.001; **, ≤ 0.01; * ≤ 0.05.

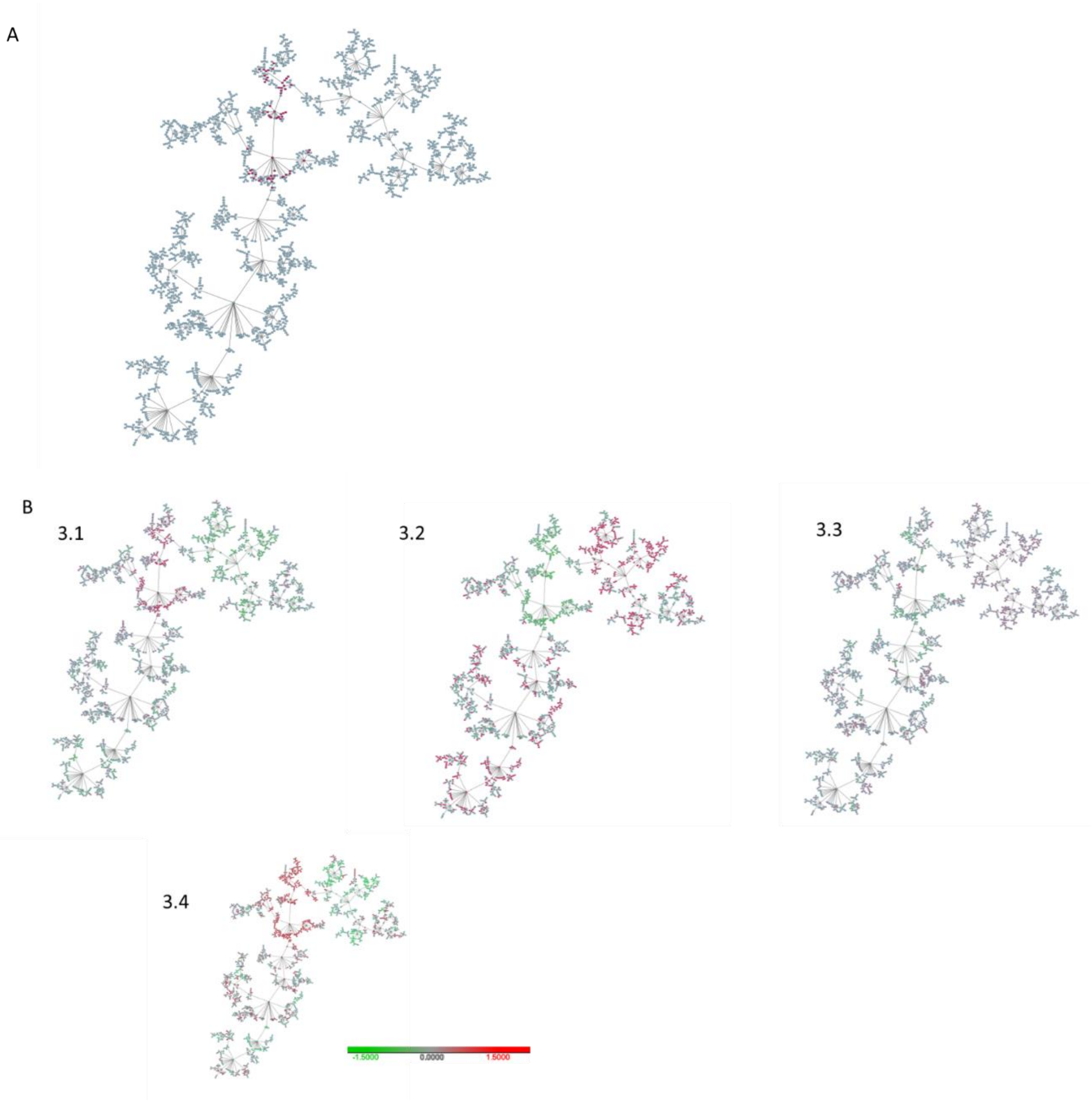
A
B
3.1
3.2
3.3
3.4
-1.5000
0.0000
1.5000

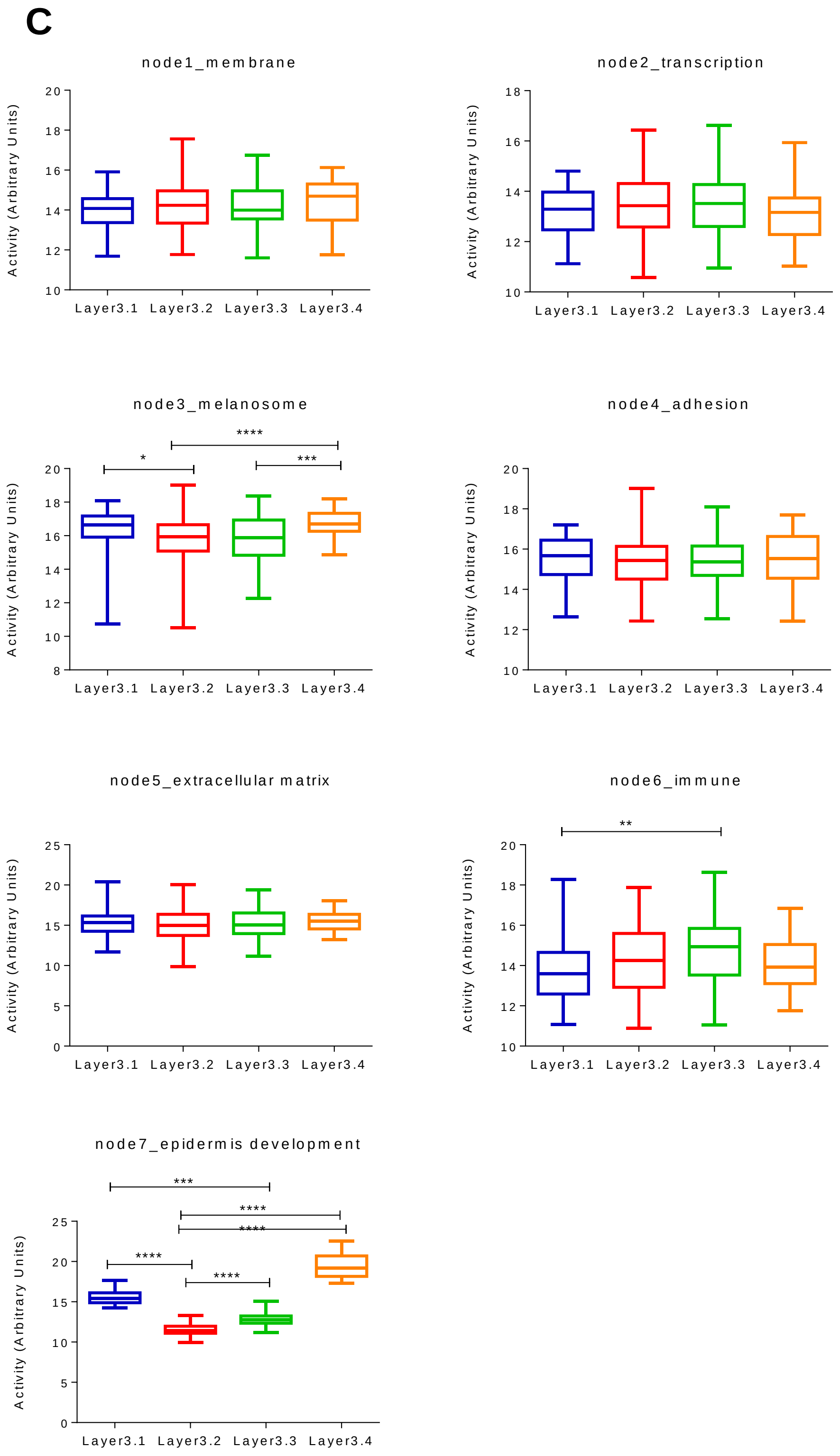

Figure S3: Third layer reflects epidermis development and keratinization in the TCGA melanoma cohort. A) Network location of the 63 genes defining Layer 3. B) Heatmap network of the four clusters of Layer 3. C) Comparison of functional node activities between defined groups. ****, ≤ 0.0001; ***, ≤ 0.001; **, ≤ 0.01; * ≤ 0.05

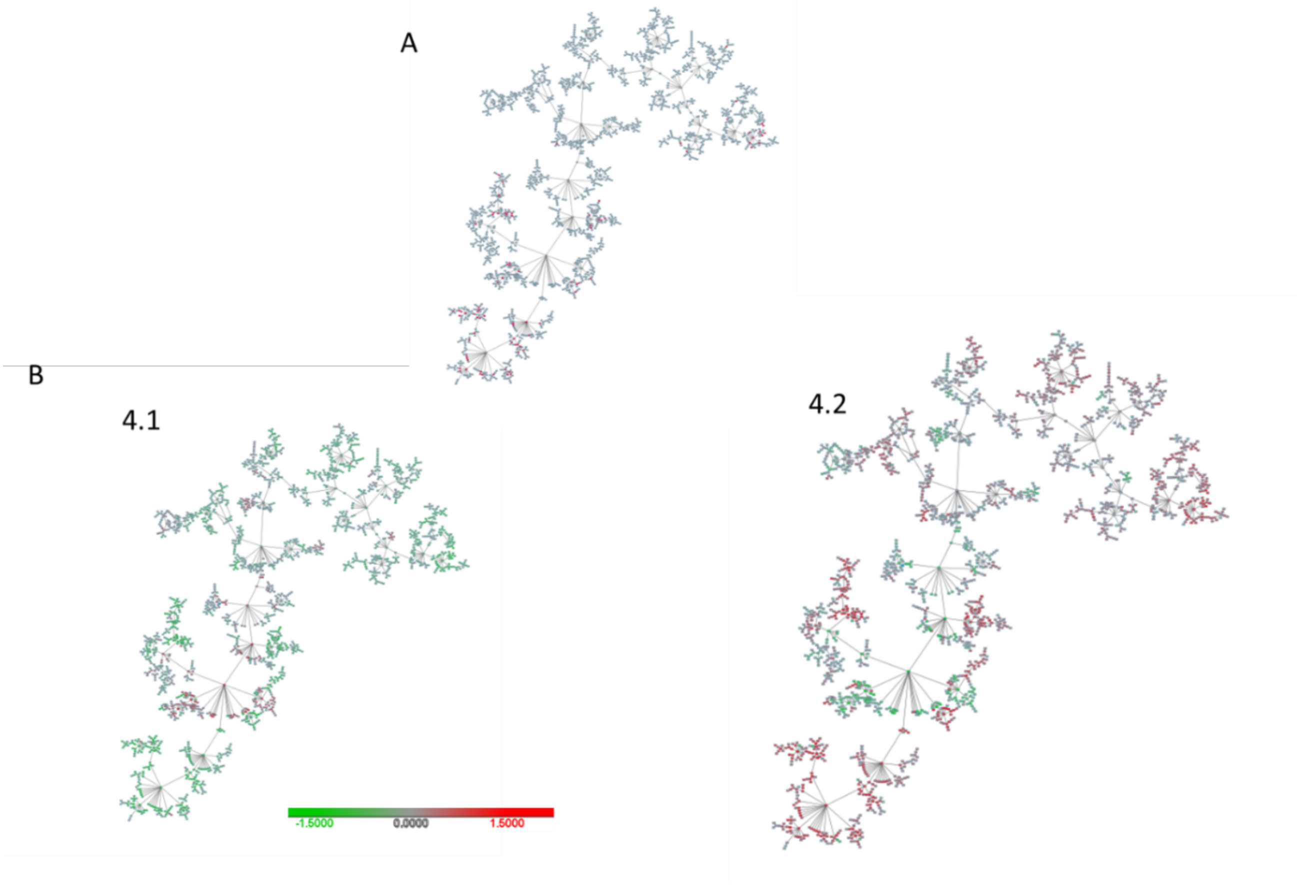

A
B
4.1
4.2
-1.5000
0.0000
1.5000

C

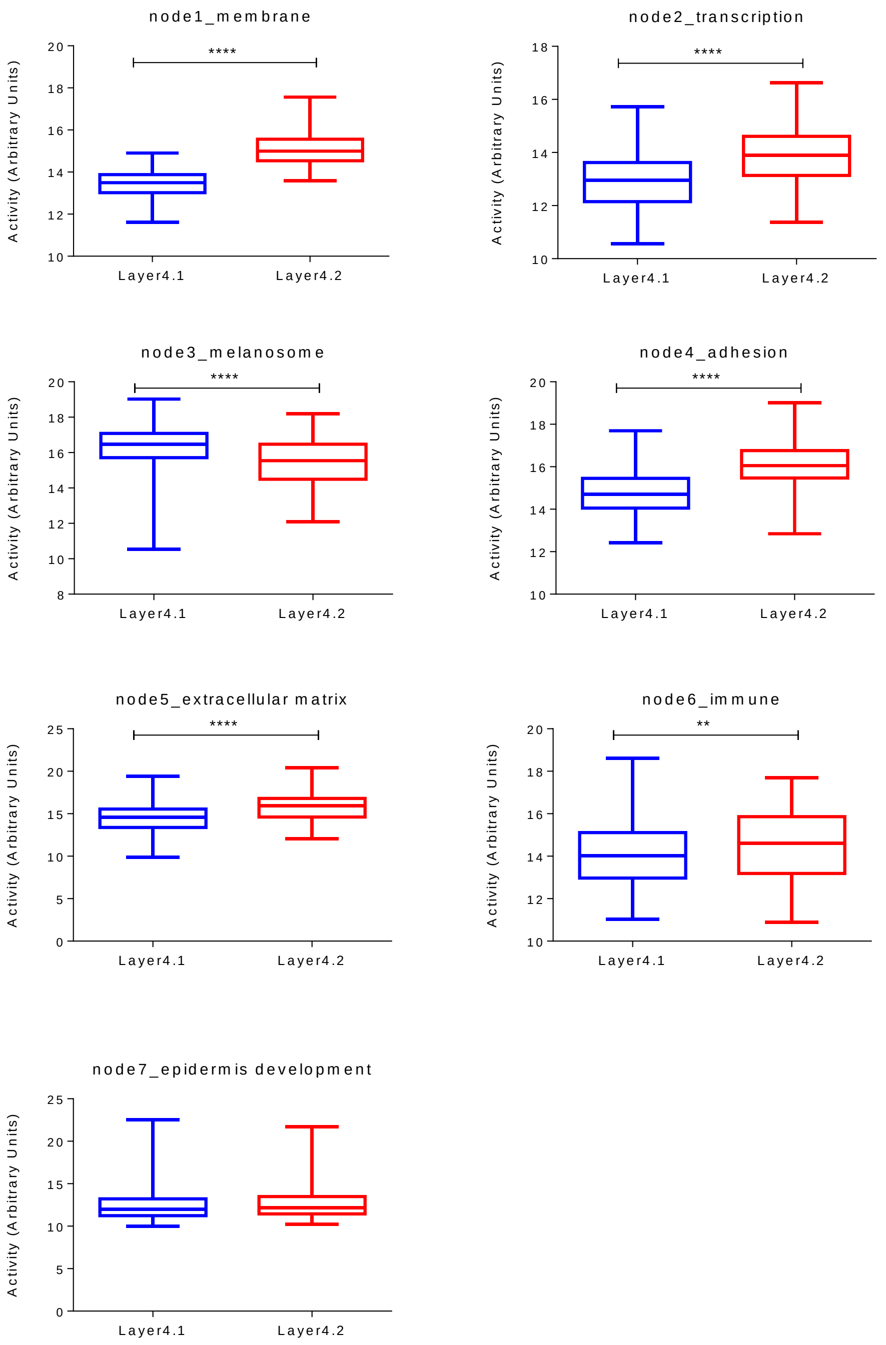

Figure S4: Fourth layer, based on extracellular matrix and adhesion genes, in the TCGA melanoma cohort. A) Network location of the 86 genes defining Layer 4. B) Heatmap network of the two groups of Layer 4. C) Comparison of functional node activities between defined groups. ****, ≤ 0.0001; ***, ≤ 0.001; **, ≤ 0.01; * ≤ 0.05.

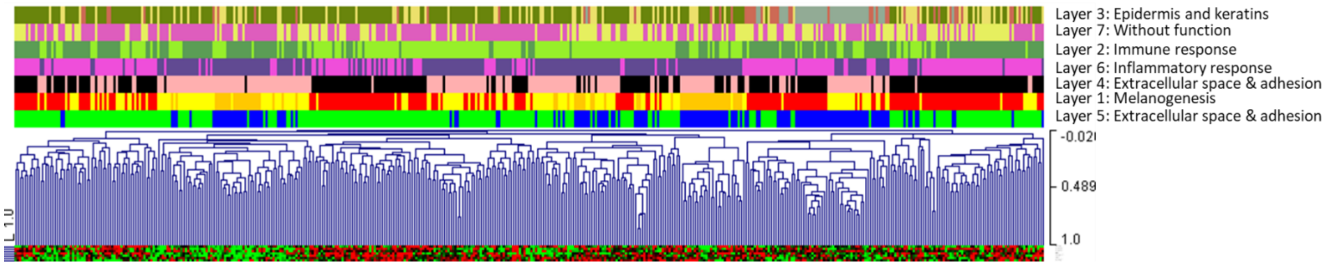

Figure S5: Hierarchical cluster grouped all classifications obtained in the layer analysis in melanoma TCGA cohort.

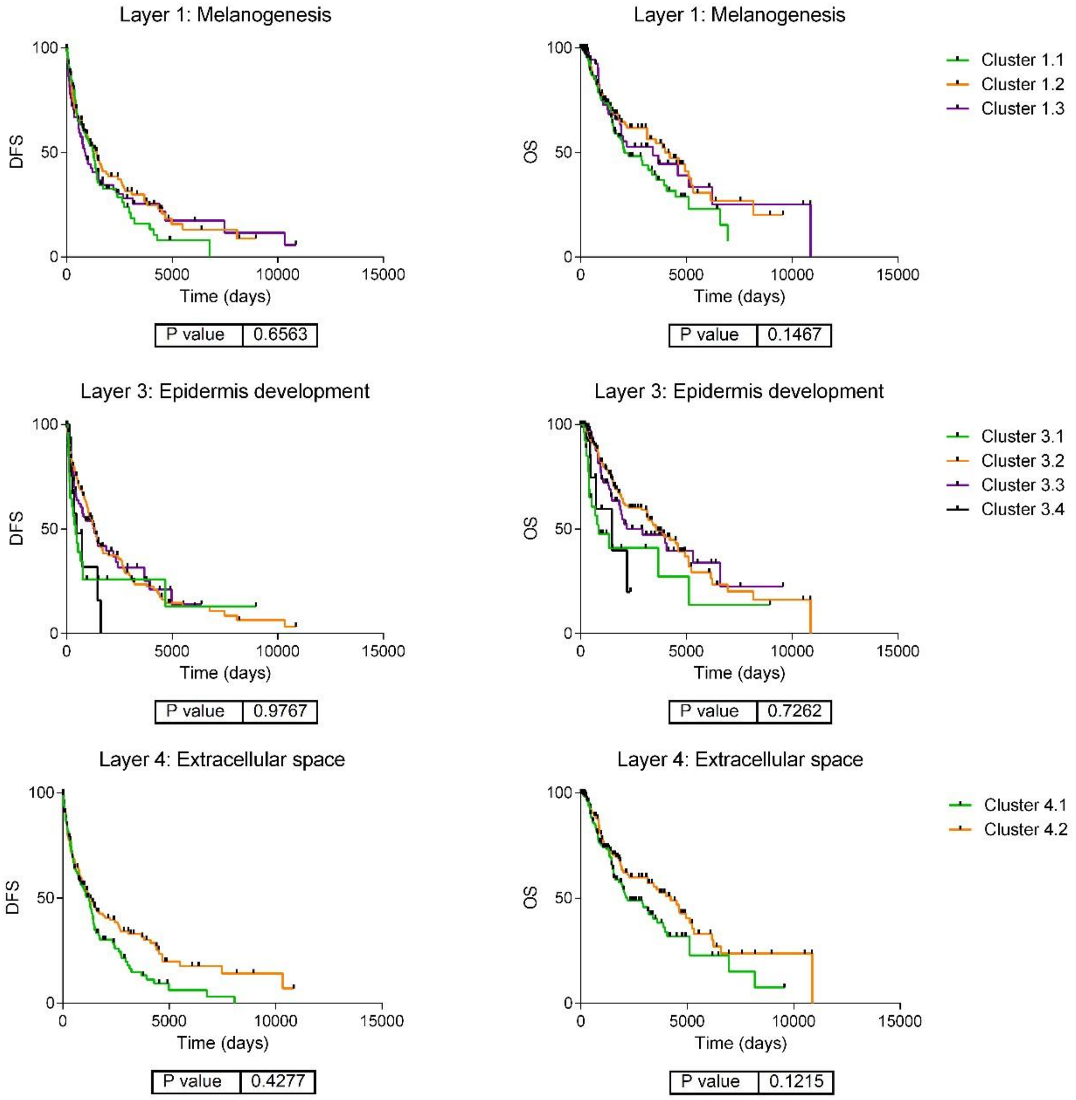

Figure S6: Survival curves according molecular layers in the TCGA melanoma cohort. DFS= disease-free survival OS= overall survival.

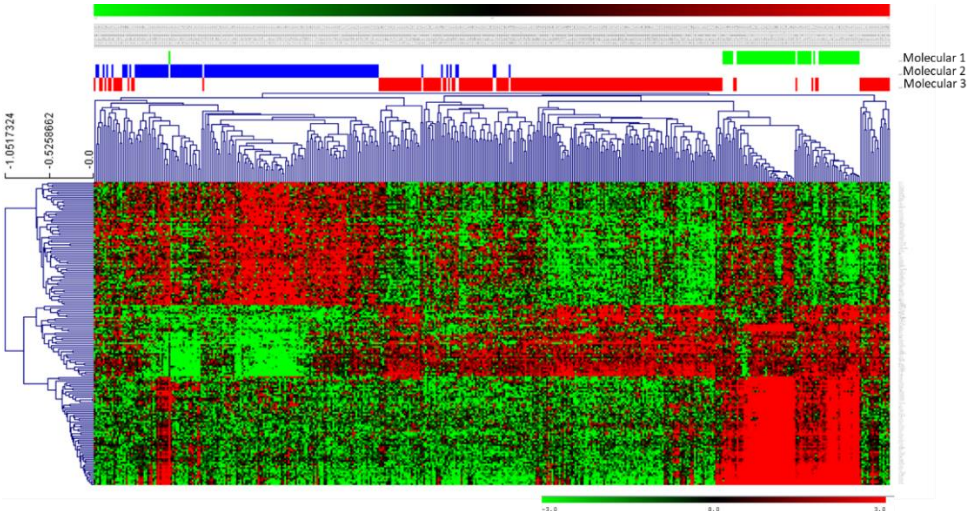

Figure S7: SAM analysis between the three molecular defined groups in TCGA melanoma cohort.

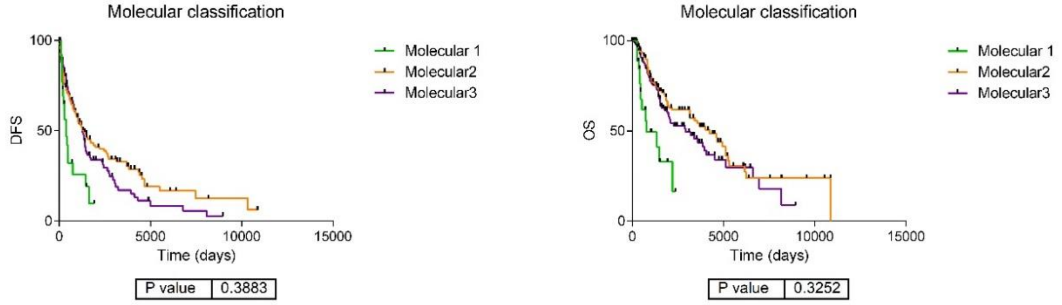

Figure S8: Survival analysis of molecular classification groups of TCGA melanoma samples. DFS= disease-free survival. OS= overall survival.

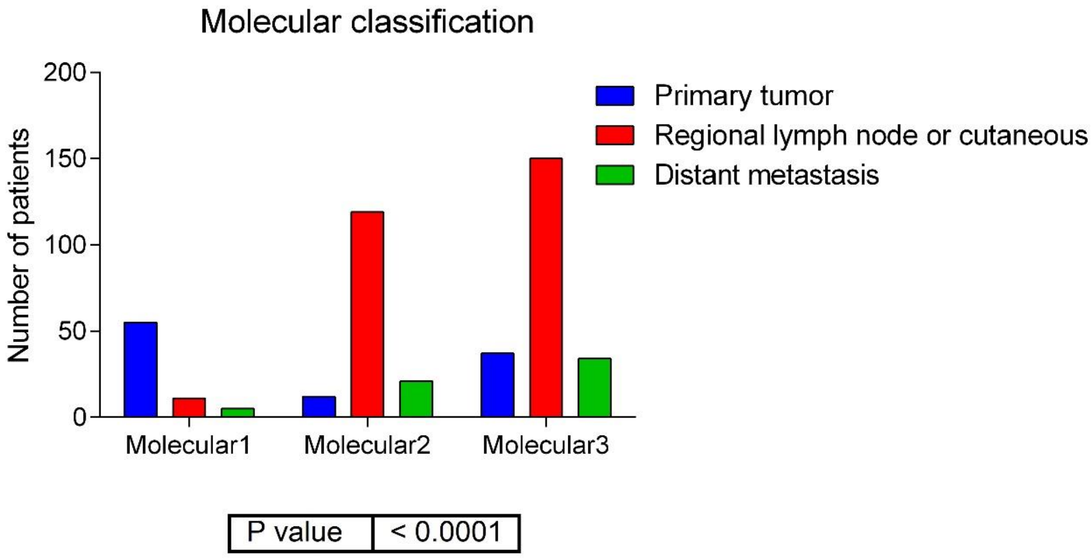

Figure S9: Distribution of primary tumors and metastases in the molecular groups in TCGA melanoma cohort.

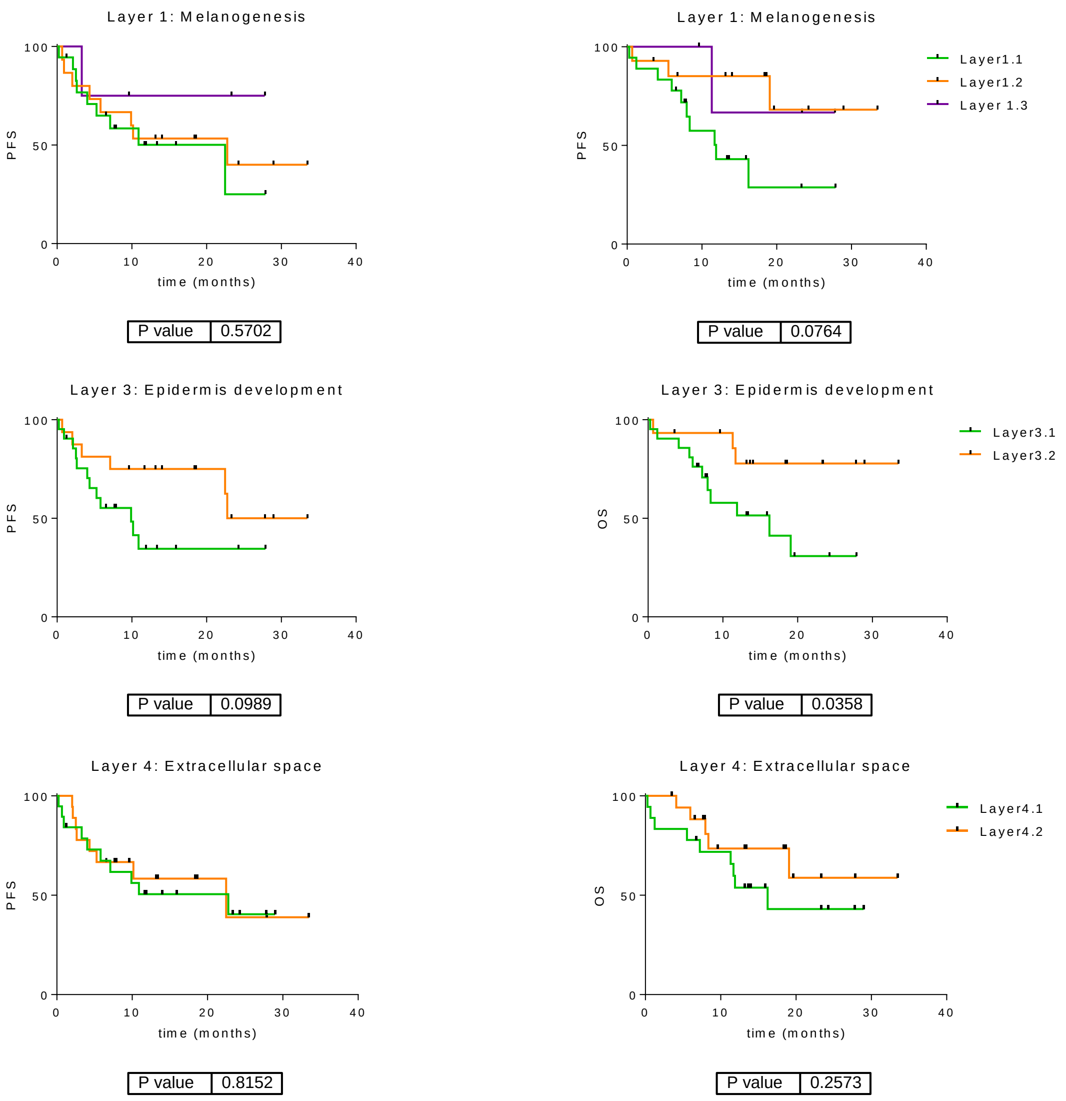

Figure S10: Survival curves according molecular layers in GEM cohort treated with PD-1 inhibitors. PFS= progression-free survival. OS= overall survival.

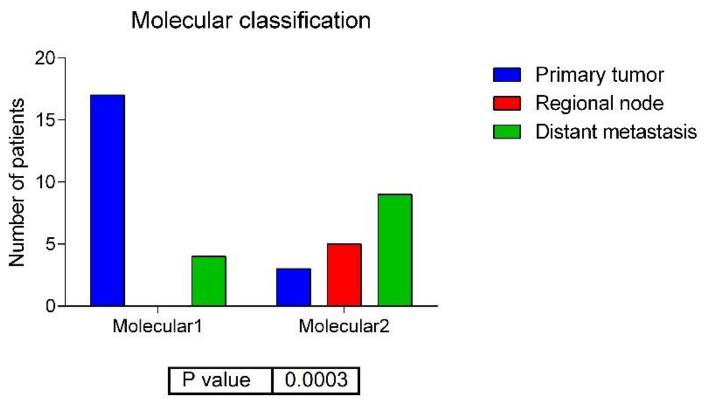

Figure S11: Distribution of primary tumors and metastases in molecular groups in GEM cohort.

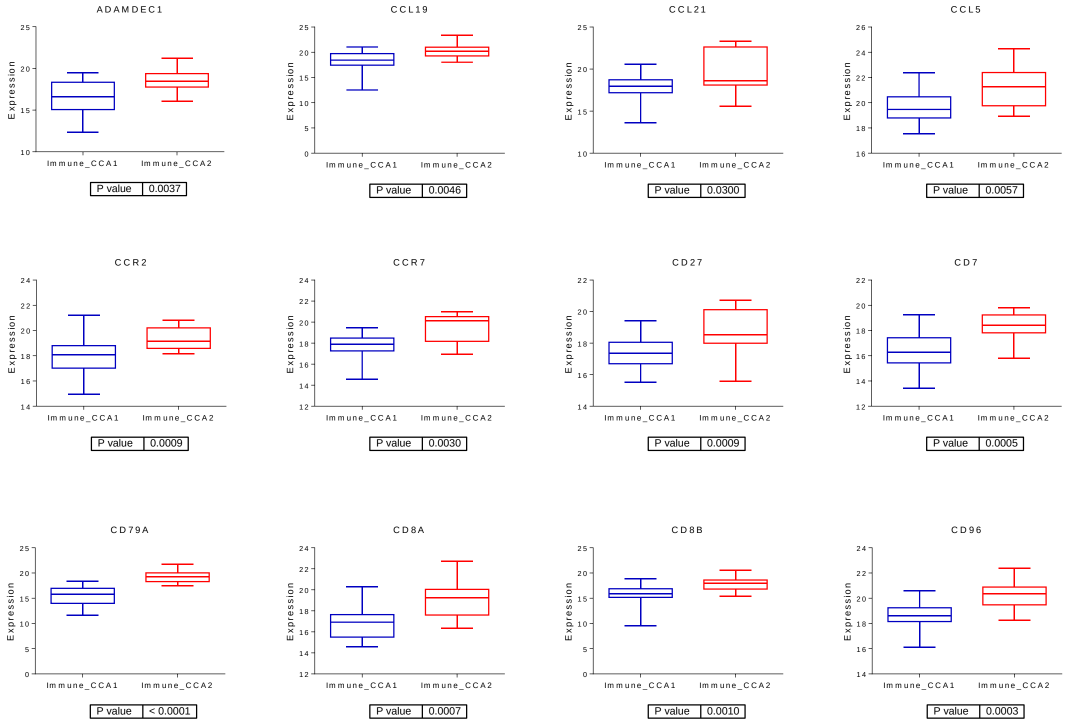

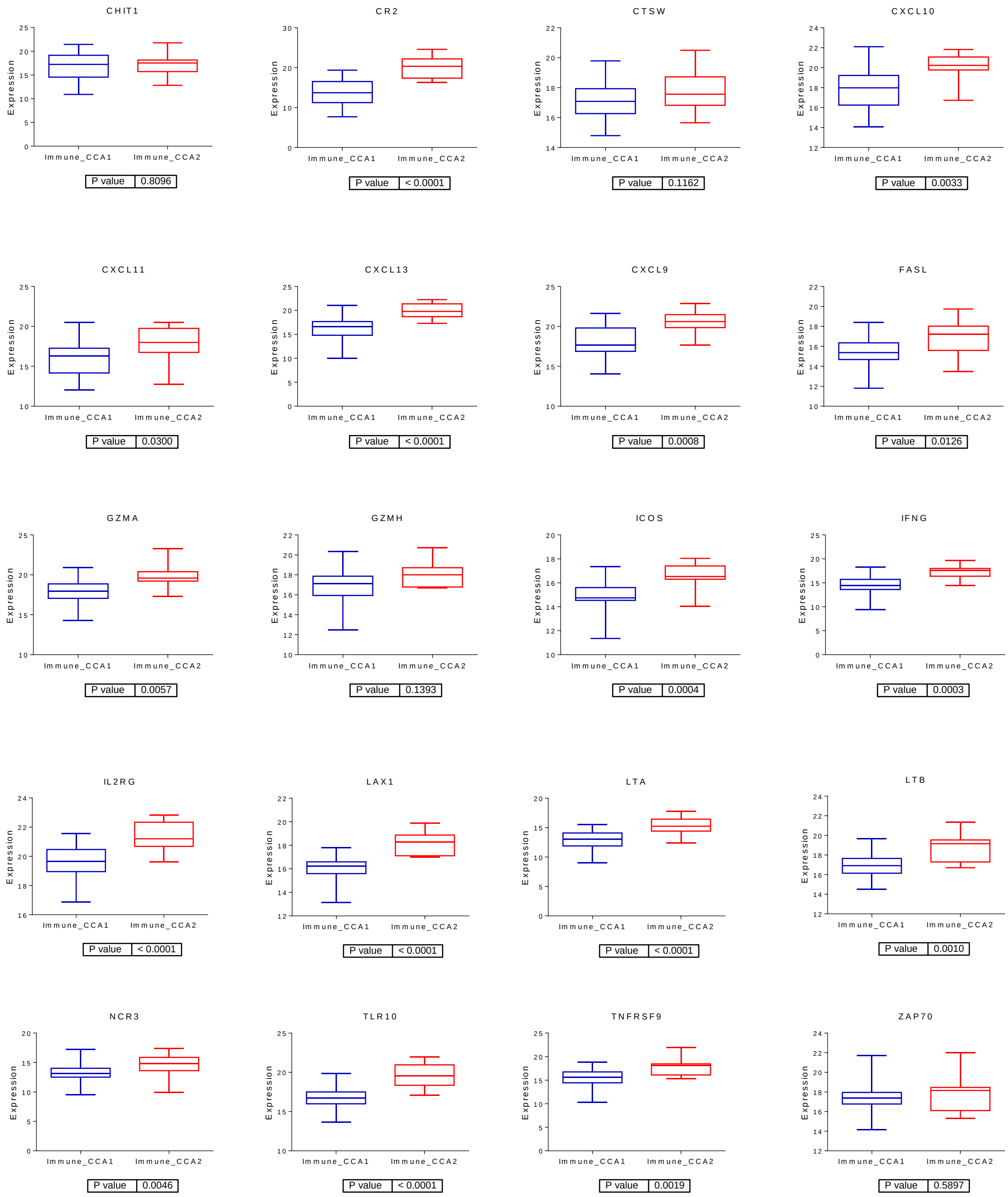

Figure S12: Immune biomarkers defined by immune layer in immunotherapy-treated GEM cohort. Expression of those genes with ontology of “immune response” contained in the immune layer in the GEM advanced melanoma cohort treated with PD1 inhibitors.
